# Uric acid-to-high-density lipoprotein cholesterol ratio and incident vascular calcification in patients with type 2 diabetes mellitus: a retrospective cohort study

**DOI:** 10.3389/fendo.2026.1964678

**Published:** 2026-10-09

**Authors:** Taoyuan He, Xin Wang, Guosheng Li, Xue Liang, Yuntong Liu, Bing Wang, Yudan Liu, Wei Zhao, Zhu Zhu, Xue Yao, Yue Xiao, Xuhan Liu

**Affiliations:** 1 Department of Endocrinology, Central Hospital of Dalian University of Technology, Dalian, Liaoning, China; 2 General Practice Ward/International Medical Center Ward, General Practice Medical Center, West China Hospital, Sichuan University, Chengdu, China; 3 Laboratory Pathology Department, Ningbo Clinical Pathology Diagnosis Center, Ningbo, China; 4 Department of Respiratory Medicine, Yanhu District People’s Hospital of Yuncheng, Yuncheng, China; 5 China-Japan Friendship Hospital (Institute of Clinical Medical Sciences), Chinese Academy of Medical Sciences and Peking Union Medical College, Beijing, China; 6 Department of Neuroendocrine Pharmacology, School of Pharmacy, China Medical University, Shenyang, China; 7 Graduate School, Dalian Medical University, Dalian, China; 8 Department of Internal Medicine, Traditional Chinese Medicine Hospital of Dalian Pulandian District, Liaoning, China; 9 Department of Endocrinology, The Second Affiliated Hospital of Dalian Medical University, Dalian Medical University, Dalian, China

**Keywords:** aortic calcification, coronary artery calcification, type 2 diabetes mellitus, uric acid-to-high-density lipoprotein cholesterol ratio, vascular calcification

## Abstract

**Background:**

The uric acid-to-high-density lipoprotein cholesterol ratio (UHR) is a composite marker that has been associated with all-cause and cardiovascular mortality in individuals with diabetes mellitus (DM). However, evidence on the association between UHR and vascular calcification (VC) remains limited and is derived primarily from cross-sectional studies of abdominal aortic calcification (AAC) in the general population. Whether UHR is associated with incident VC in patients with type 2 diabetes mellitus (T2DM) remains unclear, and longitudinal evidence in this population is lacking.

**Methods:**

This single-center retrospective cohort study included 767 hospitalized adults with T2DM who showed no evidence of VC on their initial chest computed tomography (CT). UHR was calculated using serum uric acid (SUA) and high-density lipoprotein cholesterol (HDL-C) concentrations measured during the first hospitalization. Incident coronary and/or aortic calcification was identified on follow-up chest CT examinations. The association between UHR and incident VC was evaluated using Kaplan–Meier curves with log-rank tests, multivariable Cox regression models, restricted cubic spline analysis, exploratory piecewise Cox regression, subgroup analyses, and sensitivity analyses incorporating log-transformed UHR.

**Results:**

During a median follow-up of 25 months (IQR, 11–53), 351 participants (45.8%) developed incident VC. In the fully adjusted model, each 1-standard deviation (SD) increase in UHR was associated with a 24.5% higher hazard of incident VC (hazard ratio, 1.245; 95% confidence interval, 1.117–1.387; P < 0.001). Compared with Q1, the adjusted HRs for Q2, Q3, and Q4 were 1.636, 1.921, and 1.876, respectively (P for trend < 0.001), although the quartile-specific estimates did not follow a strictly monotonic pattern. Restricted cubic spline analysis demonstrated a significant overall association between UHR and incident VC (P for overall < 0.001), whereas the test for nonlinearity was not statistically significant (P for nonlinearity = 0.058). No significant interactions were observed in prespecified subgroup analyses, and the findings remained robust across sensitivity analyses, including analysis using log-transformed UHR.

**Conclusions:**

Higher UHR was associated with a greater hazard of incident coronary and/or aortic calcification in patients with T2DM. These findings extend previous cross-sectional evidence by providing longitudinal data and warrant further evaluation of UHR as a potential marker of VC risk.

## 1 Introduction

Cardiovascular disease (CVD) encompasses a broad spectrum of disorders affecting the heart and blood vessels. According to the World Health Organization (WHO), CVD causes approximately 17.9 million deaths worldwide each year, accounting for 31% of all global deaths and representing a major public health burden (1). Vascular calcification (VC) is characterized by the pathological deposition of calcium–phosphate minerals within the vascular wall, with the osteogenic transdifferentiation of vascular smooth muscle cells (VSMCs) into osteoblast-like cells representing a key underlying mechanism (2, 3). This condition increases vascular stiffness and fragility, impairs hemodynamic function, and is associated with greater cardiovascular morbidity and mortality, including an increased risk of atherosclerosis, systolic hypertension, and coronary artery disease (CAD). These adverse effects are particularly pronounced in patients with diabetes mellitus (DM) (3–5). In patients with DM, hyperglycemia (HG) is considered a major contributor to arterial calcification. However, the ADVANCE randomized trial and its extended follow-up showed that long-term intensive glycemic control did not significantly improve macrovascular outcomes or reduce the risk of major macrovascular events or all-cause mortality. These findings suggest that the molecular mechanisms underlying arterial calcification in diabetes remain incompletely understood (4, 5).

Uric acid (UA) is the final product of purine metabolism in humans. Serum uric acid (SUA) has been independently associated with an increased risk of cardiovascular outcomes, including stroke, heart failure, coronary heart disease, and cardiovascular mortality, and may have prognostic value in both high-risk and general populations (6). Preclinical studies using animal and cellular models have shown that, both *in vivo* and *in vitro*, hyperuricemia (HUA) promotes the osteogenic transdifferentiation of VSMCs toward an osteoblast-like phenotype, thereby potentially contributing to VC (7). However, in clinical studies, the association between SUA levels and VC remains controversial (8–10). High-density lipoprotein (HDL) contributes to cardiovascular protection by promoting reverse cholesterol transport and limiting plaque formation. However, HDL dysfunction, particularly impaired cholesterol efflux capacity (CEC), may accelerate atherosclerotic progression. This dysfunction is frequently observed in metabolic disorders, including DM (11).

In recent years, the uric acid-to-high-density lipoprotein cholesterol ratio (UHR) has emerged as a novel composite metabolic marker that may integrate information on oxidative stress and anti-inflammatory capacity (12, 13). Previous cohort studies have shown that higher UHR levels are associated with an increased risk of incident ischemic heart disease (IHD) in non-diabetic individuals and with higher all-cause and cardiovascular mortality in individuals with DM or prediabetes, supporting the potential role of UHR as a cardiovascular risk marker (14, 15). Currently, direct clinical evidence linking UHR to VC remains limited and is mainly derived from cross-sectional analyses of National Health and Nutrition Examination Survey (NHANES) 2013–2014 data in the general population. These analyses generally suggest that higher UHR levels are associated with higher abdominal aortic calcification (AAC) scores and a greater likelihood of AAC (11–13, 16). However, patients with type 2 diabetes mellitus (T2DM) often have more complex disturbances in glucose and lipid metabolism and a higher burden of VC. Therefore, whether findings from the general population can be extrapolated to this high-risk group remains uncertain. Moreover, because cross-sectional designs cannot establish temporality, longitudinal studies are needed to clarify the relationship between UHR and incident VC. Therefore, we established a retrospective cohort of 767 hospitalized patients with T2DM who had no visible VC on the initial chest CT. Incident coronary and/or aortic calcification was ascertained on follow-up chest CT, and the association between UHR and incident VC risk was systematically evaluated.

## 2 Methods

### 2.1 Study design and participants

A single-center retrospective cohort was assembled from hospital records generated during routine care at the Central Hospital of Dalian University of Technology. The screening frame included 1,813 individuals with T2DM who had two or more admissions between January 2010 and January 2025. Eligibility required an age of ≥18 years, at least two chest CT examinations, absence of VC on the first CT, and availability of SUA, HDL-C, and essential clinical information from the first hospitalization. Exclusion criteria included pregnancy or lactation; malignancy; hereditary, neurological, hematologic, hepatic, renal, or severe psychiatric disorders; severe heart failure; acute or chronic infections; acute complications; disorders affecting calcium–phosphorus metabolism; use within the preceding 12 months of drugs known to influence uric acid metabolism (including allopurinol, febuxostat, benzbromarone, or probenecid); and insufficient clinical or follow-up information. Application of these criteria yielded a final analytic cohort of 767 participants (**Figure 1**). For categorical analyses, baseline UHR was divided into quartiles. Baseline characteristics were compared according to whether VC occurred during follow-up.

**Figure 1:**
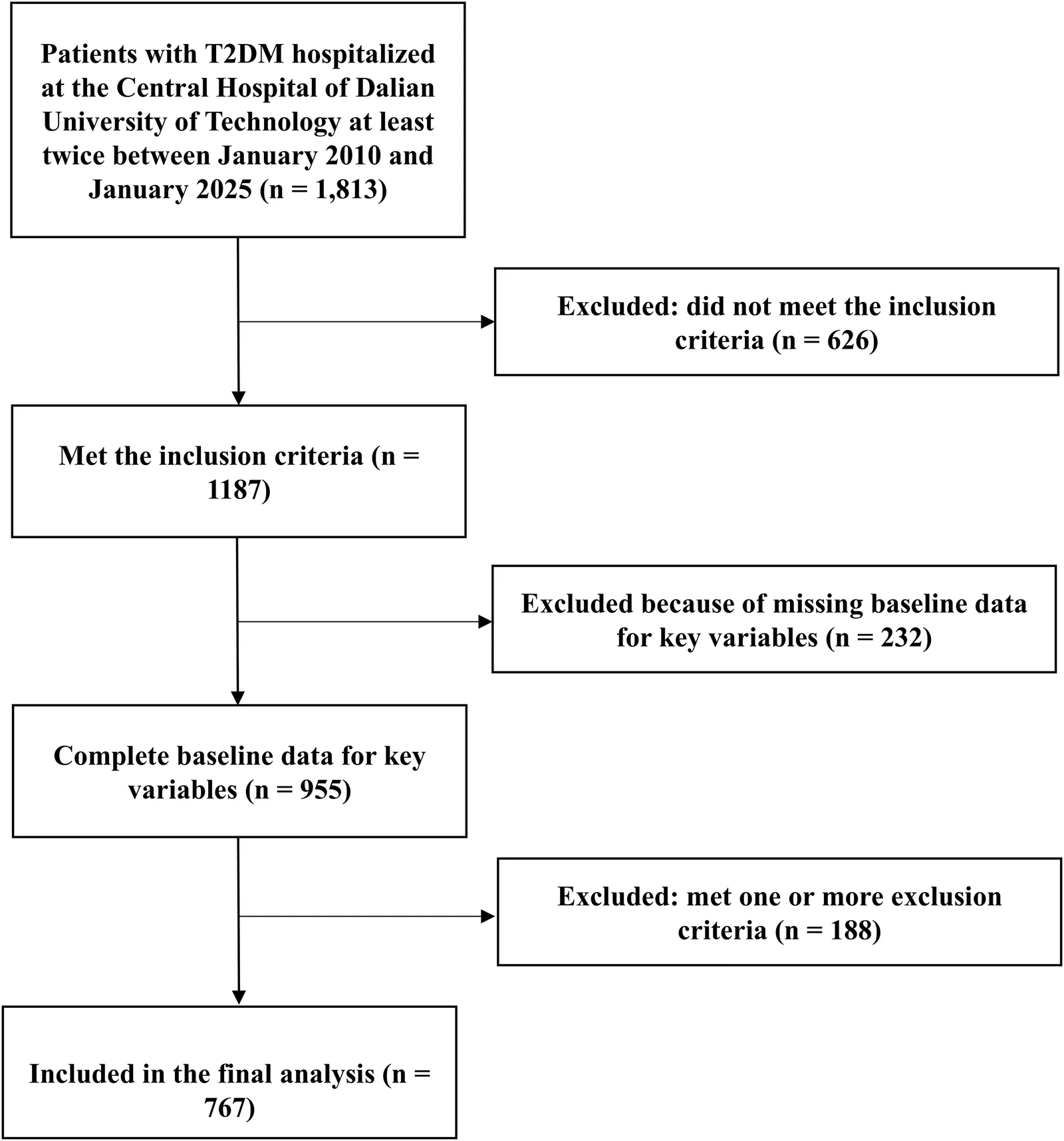
Flowchart of participant selection.

### 2.2 Data collection and laboratory measurements

Baseline variables were retrieved from the YiduCloud Electronic Medical Record Retrieval System and included demographic characteristics, smoking and alcohol use, medical history, family history, DM duration, medication use, anthropometric and hemodynamic measurements, and laboratory data. Medication variables included insulin therapy and lipid-lowering therapy. Laboratory measurements included fasting plasma glucose (FPG), glycated hemoglobin (HbA1c), triglycerides (TG), high-density lipoprotein cholesterol (HDL-C), SUA, hemoglobin, alkaline phosphatase (ALP), calcium, magnesium, and creatinine. Detailed class-specific information on antihypertensive and glucose-lowering therapies, including the use of diuretics and SGLT2 inhibitors, was not consistently available throughout the study period and therefore could not be comprehensively evaluated. Family history was defined as a self-reported history of DM and/or cardiovascular disease in first-degree relatives, including parents and siblings. HbA1c was quantified by high-performance liquid chromatography on an HLC-723G8 analyzer (TOSOH, Japan); the remaining biochemical assays were performed on an ADVIA 2400 analyzer (Siemens, Germany).

### 2.3 Disease definitions

Hypertension was defined according to the *Chinese Guidelines for the Prevention and Treatment of Hypertension (2024 revision)* (17); participants with a documented history of hypertension or receiving antihypertensive therapy were also classified as having hypertension. T2DM was defined according to the *Guidelines on Primary Healthcare for Type 2 Diabetes in China, 2025* (18); participants with a documented history of T2DM or receiving glucose-lowering therapy were also classified as having T2DM. The estimated glomerular filtration rate (eGFR) was calculated using the Chronic Kidney Disease Epidemiology Collaboration (CKD-EPI) equation (19).

### 2.4 Calculation of UHR

UHR was calculated as [SUA (mg/dL)/HDL-C (mg/dL)] × 100. Multiplication by 100 was used solely as a scaling factor to facilitate presentation and interpretation. SUA and HDL-C were originally measured in μmol/L and mmol/L, respectively, and were converted to mg/dL before calculation of UHR as follows: SUA (mg/dL) = SUA (μmol/L) ÷ 59.48 and HDL-C (mg/dL) = HDL-C (mmol/L) × 38.67.

### 2.5 VC assessment and follow-up

Chest CT examinations were performed using a GE HD750 64-slice CT system. Vascular calcification was assessed qualitatively on mediastinal-window images based on the presence of visually apparent calcified foci or plaques along the coronary arteries and/or aortic wall. No prespecified attenuation threshold, minimum lesion size, or quantitative calcium-scoring criterion was applied. Incident VC was defined as newly detected coronary and/or aortic calcification on follow-up CT in a participant whose initial CT showed no visible VC. Incident VC events were classified by vascular territory as coronary artery calcification (CAC), aortic calcification, or combined coronary and aortic calcification. The study outcome was defined as the first detection of newly developed visible VC during follow-up; calcification burden, severity, and subsequent progression were not quantitatively assessed. Two trained attending radiologists independently interpreted the eligible CT examinations without access to participants’ clinical information. Inter-reader concordance at the initial assessment was summarized using Cohen’s kappa coefficient with a 95% CI; discordant interpretations were resolved through consensus review. Follow-up time accrued from the first hospitalization until the CT examination that first documented incident VC. Participants who remained free of VC were censored at their last available chest CT.

To further characterize the clinical context of CT acquisition, two investigators independently classified the indications for both baseline and outcome-defining follow-up CT examinations into five categories: respiratory symptoms or suspected pulmonary disease, follow-up of previously identified thoracic abnormalities, preoperative or perioperative assessment, routine or other non-acute clinical evaluation, and other or unclear indications. Disagreements were resolved by consensus, and the distributions of CT indications were compared between participants with and without incident VC. In addition, because CT examinations were performed according to clinical indications rather than a predefined imaging protocol, the intervals between baseline and follow-up CT examinations were not standardized and varied across participants.

### 2.6 Statistical analysis

Statistical analyses were performed using R software (version 4.5.3) and Zstats v1.0 (www.zstats.net). The normality of continuous variables was assessed using the Shapiro–Wilk test. Normally distributed variables were expressed as mean ± standard deviation (SD), skewed variables as median (interquartile range [IQR]), and categorical variables as number (percentage). Baseline characteristics were compared between participants with and without incident VC using the independent-samples t test, Mann–Whitney U test, chi-square test, or Fisher’s exact test, as appropriate.

UHR was modeled both continuously (per 1-SD increase) and categorically using quartiles: Q1 (≤10.692), Q2 (>10.692 to ≤13.465), Q3 (>13.465 to ≤16.495), and Q4 (>16.495). Kaplan–Meier estimates were used to characterize VC-free survival according to UHR quartile, with between-group differences tested using the log-rank test. Cox proportional hazards models were fitted to estimate hazard ratios (HRs) and 95% confidence intervals (CIs) for incident VC in relation to UHR. Model 1 was unadjusted. Model 2 was adjusted for age and sex. Model 3 was further adjusted for alcohol use, smoking status, family history, hypertension, body mass index (BMI), FPG, HbA1c, diabetes duration, and eGFR. Model 4 was additionally adjusted for TG, ALP, magnesium, Ca, insulin therapy, and lipid-lowering therapy. To examine whether the association between UHR and incident VC was attributable to either of its components, separate Cox proportional hazards models were fitted for SUA and HDL-C using the same covariate adjustment strategy as for UHR. UHR, SUA, and HDL-C were standardized and analyzed per 1-SD increase to facilitate comparison of effect estimates across markers measured on different scales. Linear trends across UHR quartiles were evaluated by assigning the median UHR value of each quartile to participants within that quartile and entering this variable as a continuous term in the Cox regression models. In addition, the overall association between UHR quartiles and incident VC was assessed using a global likelihood-ratio test with three degrees of freedom. The proportional hazards assumption was assessed using Schoenfeld residual tests and corresponding plots.

To characterize the potential non-linear relationship between UHR and incident VC, restricted cubic spline (RCS) functions were fitted with adjustment for Model 4 covariates, with knots placed at the 5th, 35th, 65th, and 95th percentiles. An exploratory two-piecewise Cox proportional hazards model was subsequently fitted to explore potential changes in the association pattern suggested by quartile-specific estimates. A data-driven breakpoint was estimated, and associations between UHR and incident VC below and above the breakpoint were evaluated separately. Model fit was compared using a likelihood-ratio test, and the uncertainty of the breakpoint estimate was evaluated using 1,000 iterations of nonparametric bootstrap resampling. The association was further examined within strata defined by age (<65 or ≥65 years), sex, BMI (<25 or ≥25 kg/m²), smoking status, and hypertension. Within each subgroup, models included all Model 4 covariates except the corresponding stratification variable. Effect modification was evaluated by adding an interaction term between UHR and each subgroup variable to the fully adjusted model.

Three sensitivity analyses were conducted to assess the robustness of the findings. First, participants who developed VC within the first year of follow-up were excluded to minimize the potential impact of reverse causation. Second, participants receiving lipid-lowering therapy at the first hospitalization were excluded to evaluate whether the observed association remained robust after accounting for potential treatment-related effects. Third, because UHR exhibited a non-normal distribution, it was natural-log transformed and re-evaluated in an additional sensitivity analysis. The log-transformed UHR was standardized before inclusion in the Cox models, allowing the hazard ratio to be interpreted per 1-SD increase in log-transformed UHR. Cox proportional hazards models were refitted using the same covariate adjustment strategy as in the primary analysis. Statistical significance was defined using a two-sided P value <0.05.

## 3 Results

### 3.1 Baseline characteristics and development of incident VC during follow-up

A total of 767 patients with T2DM and no VC at baseline were included; 351 (45.8%) developed incident VC during follow-up, whereas 416 remained free of VC. Among the 351 patients who developed incident VC, 72 (20.5%) had CAC only, 113 (32.2%) had aortic calcification only, and 166 (47.3%) had both coronary and aortic calcification. Overall, incident CAC occurred in 238 patients (31.0%), whereas incident aortic calcification occurred in 279 (36.4%). The median follow-up duration was 25 months (IQR, 11–53; range, 1–173 months). The two radiologists showed near-perfect agreement in the assessment of VC on CT images, with a Cohen’s kappa coefficient of 0.807 (95% CI, 0.765–0.849). The mean age of the participants was 57.73 ± 11.36 years, and 415 (54.1%) were female.

Compared with participants who remained free of VC, those who developed VC were older (59.80 ± 10.22 vs. 55.99 ± 11.99 years, P < 0.001) and had a higher prevalence of smoking (39.9% vs. 32.0%, P = 0.023) and a higher BMI (26.65 ± 3.56 vs. 25.75 ± 3.62 kg/m², P < 0.001). They also had higher diastolic blood pressure (BP) (84.00 [80.00–90.00] vs. 80.00 [78.75–90.00] mmHg, P = 0.023), FPG (9.02 [7.63–11.07] vs. 7.95 [7.45–10.13] mmol/L, P < 0.001), and HbA1c (8.69 ± 1.11% vs. 8.44 ± 1.03%, P = 0.001), but lower eGFR (93.53 [89.83–97.77] vs. 95.59 [89.24–101.03] mL/min per 1.73 m², P = 0.014). Serum uric acid was also higher in participants who developed VC than in those who remained free of VC (344.02 ± 83.07 vs. 303.76 ± 75.87 μmol/L, P < 0.001). UHR was likewise higher in the VC group than in the non-VC group (14.06 [11.40–17.35] vs. 12.55 [10.03–16.03], P < 0.001).

No significant differences were observed between the groups in sex, alcohol use, family history, hypertension, insulin therapy, lipid-lowering therapy, DM duration, systolic BP, heart rate, TG, HDL-C, hemoglobin, ALP, serum calcium, or serum magnesium (all P > 0.05; **Table 1**). The distributions of indications for both baseline and outcome-defining follow-up chest CT examinations did not differ significantly between participants with and without incident VC (P = 0.759 and 0.298, respectively). Interrater agreement for indication classification was high for both baseline and follow-up CT examinations (Cohen’s κ = 0.886 and 0.881, respectively; **Supplementary Tables S1**, **S2**).

**Table 1:** Baseline characteristics of study participants.

Variables	Total (n = 767)	Non-VC (n = 416)	VC (n = 351)	*P* value
Age (years)	57.73 ± 11.36	55.99 ± 11.99	59.80 ± 10.22	<0.001
Sex (%)				0.195
Female	415 (54.1)	234 (56.3)	181 (51.6)	
Male	352 (45.9)	182 (43.8)	170 (48.4)	
Alcohol use (%)				0.720
No	562 (73.3)	307 (73.8)	255 (72.7)	
Yes	205 (26.7)	109 (26.2)	96 (27.4)	
Smoking (%)				0.023
No	494 (64.4)	283 (68.0)	211 (60.1)	
Yes	273 (35.6)	133 (32.0)	140 (39.9)	
Family history (%)				0.196
No	444 (57.9)	232 (55.8)	212 (60.4)	
Yes	323 (42.1)	184 (44.2)	139 (39.6)	
Hypertension (%)				0.911
No	685 (89.3)	372 (89.4)	313 (89.2)	
Yes	82 (10.7)	44 (10.6)	38 (10.8)	
Insulin therapy (%)				0.401
No	743 (96.9)	405 (97.4)	338 (96.3)	
Yes	24 (3.1)	11 (2.6)	13 (3.7)	
Lipid-lowering therapy (%)				0.555
No	754 (98.3)	410 (98.6)	344 (98.0)	
Yes	13 (1.7)	6 (1.4)	7 (2.0)	
DM duration (years)	6.00 (3.00, 9.00)	6.00 (3.00, 9.00)	6.00 (3.00, 9.00)	0.227
BMI (kg/m2)	26.16 ± 3.62	25.75 ± 3.62	26.65 ± 3.56	<0.001
Systolic BP (mmHg)	139.00 (125.00, 150.00)	135.00 (124.00, 150.00)	140.00 (130.00, 150.00)	0.094
Diastolic BP (mmHg)	81.00 (80.00, 90.00)	80.00 (78.75, 90.00)	84.00 (80.00, 90.00)	0.023
Heart rate (beats/min)	78.00 (74.00, 80.00)	78.00 (74.00, 82.00)	78.00 (74.00, 80.00)	0.302
eGFR (mL/min per 1.73 m2)	94.83 (89.49, 99.12)	95.59 (89.24, 101.03)	93.53 (89.83, 97.77)	0.014
FPG (mmol/L)	8.20 (7.55, 10.71)	7.95 (7.45, 10.13)	9.02 (7.63, 11.07)	<0.001
HbA1c (%)	8.55 ± 1.07	8.44 ± 1.03	8.69 ± 1.11	0.001
TG (mmol/L)	1.63 (1.19, 2.33)	1.63 (1.19, 2.30)	1.63 (1.18, 2.35)	0.702
HDL-C (mmol/L)	1.03 (0.89, 1.17)	1.02 (0.88, 1.17)	1.04 (0.90, 1.17)	0.491
SUA (μmol/L)	322.18 ± 81.70	303.76 ± 75.87	344.02 ± 83.07	<0.001
Hemoglobin (g/L)	139.93 ± 15.93	140.42 ± 16.82	139.34 ± 14.80	0.341
ALP (U/L)	74.00 (63.00, 90.00)	74.00 (63.00, 90.00)	75.00 (62.00, 89.17)	0.831
Serum calcium (mmol/L)	2.34 ± 0.11	2.34 ± 0.11	2.34 ± 0.10	0.597
Serum magnesium (mmol/L)	0.89 ± 0.07	0.89 ± 0.07	0.88 ± 0.07	0.213
UHR	13.46 (10.69, 16.49)	12.55 (10.03, 16.03)	14.06 (11.40, 17.35)	<0.001

Data are presented as mean ± SD, median (IQR), or n (%). P values correspond to comparisons of baseline characteristics between participants who developed VC and those who remained free of VC during follow-up. Family history was defined as a self-reported history of DM and/or cardiovascular disease in first-degree relatives, including parents and siblings.

### 3.2 VC-free survival according to UHR quartiles

Kaplan–Meier analysis showed significant differences in VC-free survival across UHR quartiles (log-rank P < 0.001; **Figure 2**). Participants in Q1 generally had the highest VC-free survival probability for most of the follow-up period, whereas the survival curves for Q2–Q4 were lower and showed substantial overlap.

**Figure 2:**
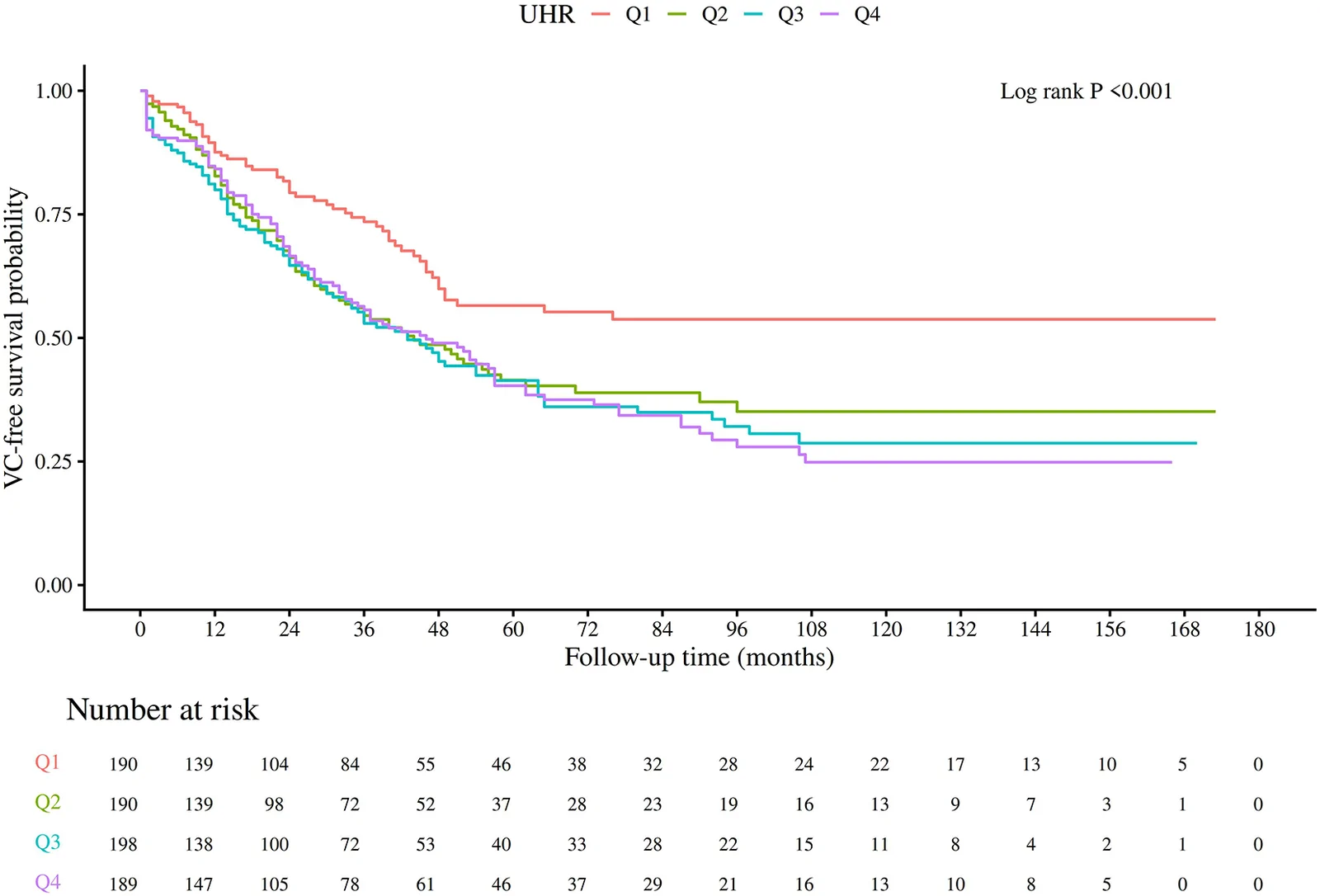
Kaplan–Meier curves of VC-free survival according to UHR quartiles. Differences among the curves were assessed using the log-rank test. Numbers at risk are shown below the curves.

### 3.3 Association between UHR and incident VC

Cox proportional hazards regression analyses showed that higher UHR was associated with a greater hazard of incident VC. When modeled as a continuous variable, each 1-SD increase in UHR was associated with a 24.5% higher hazard of incident VC in the fully adjusted model (Model 4: HR, 1.245; 95% CI, 1.117–1.387; P < 0.001). The association remained statistically significant across all four models.

When UHR was categorized into quartiles, participants in Q2, Q3, and Q4 had higher risks of incident VC compared with those in Q1 in the fully adjusted model, with HRs of 1.636 (95% CI, 1.166–2.295; P = 0.004), 1.921 (95% CI, 1.379–2.675; P < 0.001), and 1.876 (95% CI, 1.343–2.620; P < 0.001), respectively. The overall association of UHR quartiles with incident VC was statistically significant (P for overall < 0.001). A significant positive trend was observed when the median UHR value of each quartile was modeled as a continuous variable (P for trend < 0.001). However, the HR for Q4 was slightly lower than that for Q3, suggesting that the quartile-specific associations did not follow a strictly monotonic pattern (**Table 2**).

**Table 2:** Associations between UHR and incident VC.

Variable	Model 1		Model 2		Model 3		Model 4	
	HR (95% CI)	*P* value	HR (95% CI)	*P* value	HR (95% CI)	*P* value	HR (95% CI)	*P* value
UHR								
Per 1 SD increase	1.194 (1.080–1.319)	<0.001	1.231 (1.113–1.362)	<0.001	1.235 (1.112–1.371)	<0.001	1.245 (1.117–1.387)	<0.001
Quartiles 1	Ref		Ref		Ref		Ref	
Quartiles 2	1.694 (1.214–2.362)	0.002	1.786 (1.278–2.494)	<0.001	1.668 (1.190–2.337)	0.003	1.636 (1.166–2.295)	0.004
Quartiles 3	1.867 (1.349–2.585)	<0.001	2.022 (1.457–2.806)	<0.001	1.940 (1.396–2.696)	<0.001	1.921 (1.379–2.675)	<0.001
Quartiles 4	1.837 (1.331–2.536)	<0.001	1.992 (1.439–2.757)	<0.001	1.876 (1.351–2.606)	<0.001	1.876 (1.343–2.620)	<0.001
*P* for overall		<0.001		<0.001		<0.001		<0.001
*P* for trend		<0.001		<0.001		<0.001		<0.001

Model 1 was unadjusted. Model 2 was adjusted for age and sex. Model 3 was further adjusted for alcohol use, smoking status, family history, hypertension, BMI, FPG, HbA1c, DM duration, and eGFR. Model 4 was additionally adjusted for TG, ALP, Mg, Ca, insulin therapy, and lipid-lowering therapy.

Global tests based on Schoenfeld residuals indicated no violation of the proportional hazards assumption when UHR was modeled as either a continuous variable or quartiles (P = 0.374 and P = 0.540, respectively; **Supplementary Figures S1**, **S2**).

Additional component analyses showed that the association between SUA and incident VC was attenuated after progressive multivariable adjustment and was no longer statistically significant in the fully adjusted model. HDL-C was likewise not significantly associated with incident VC in the fully adjusted model. In contrast, the association between UHR and incident VC remained statistically significant after full adjustment (**Supplementary Table S3**).

### 3.4 RCS and exploratory threshold analysis

RCS analysis demonstrated a significant overall association between UHR and the hazard of incident VC (P for overall <0.001), whereas the test for nonlinearity was not statistically significant (P for nonlinearity = 0.058). The spline curve suggested a potential deviation from linearity, with an initial increase in risk followed by attenuation in the intermediate range and a subsequent increase at higher UHR levels (**Figure 3**). To further characterize this potential pattern, an exploratory two-piecewise Cox proportional hazards model adjusted for Model 4 covariates was fitted, identifying a data-derived breakpoint at a UHR value of 11.388. Below the breakpoint, each 1-unit increase in UHR was associated with a higher hazard of incident VC (HR, 1.199; 95% CI, 1.068–1.347), whereas the magnitude of the association was reduced above the breakpoint (HR, 1.026; 95% CI, 0.996–1.057). The likelihood-ratio test comparing the two-piecewise and single-linear models indicated a better model fit for the two-piecewise model (P = 0.014). However, bootstrap analysis revealed substantial uncertainty in the breakpoint estimate (median, 11.415; 95% CI, 9.235–20.806), and the bootstrap confidence interval for the change in slope included zero (−0.160; 95% CI, −0.350 to 0.121), suggesting limited stability of the identified breakpoint. These findings suggest a potential attenuation of the association at higher UHR levels; however, the identified threshold should be interpreted cautiously given the uncertainty of the breakpoint estimate.

**Figure 3:**
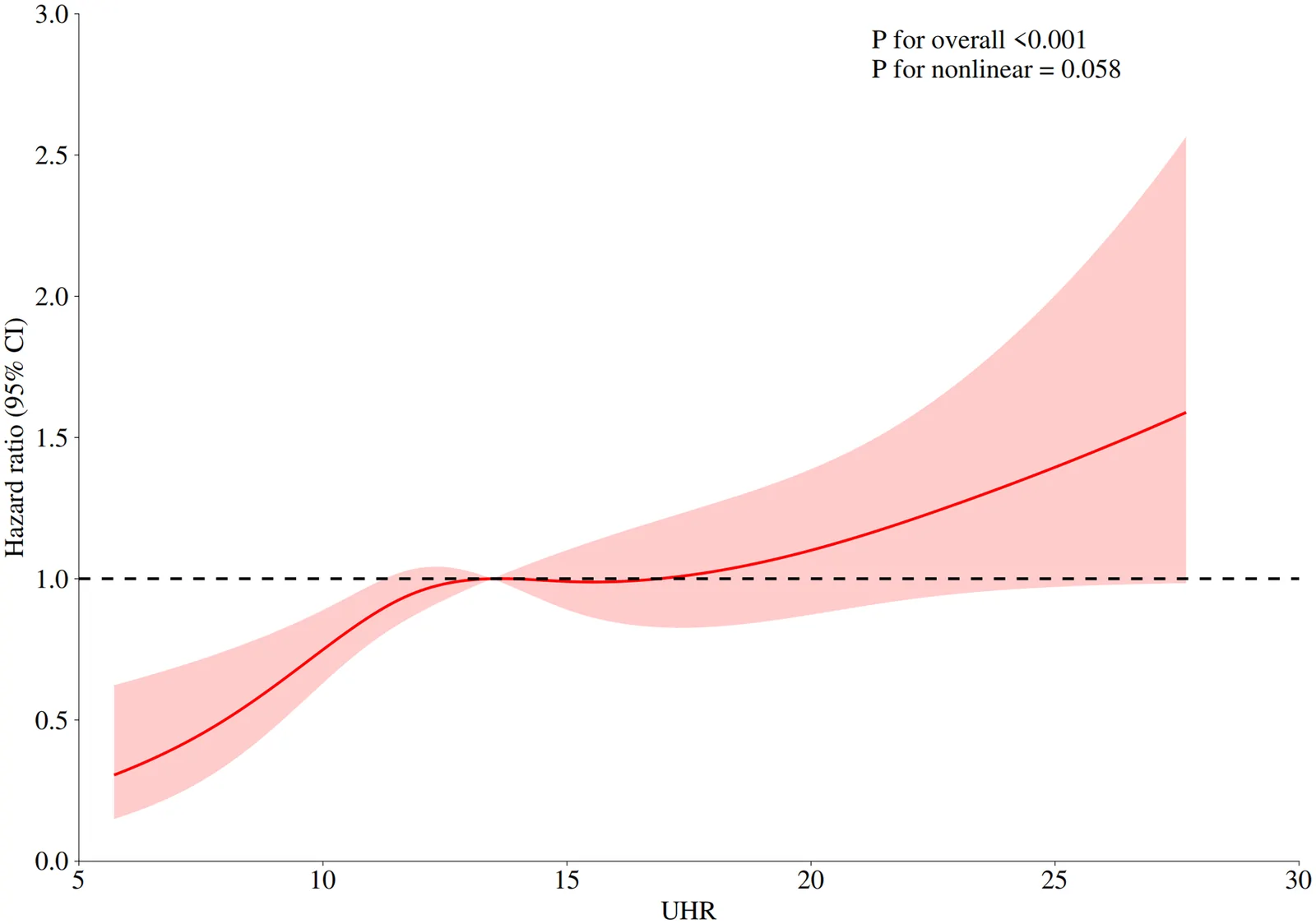
RCS analysis of the association between UHR and incident VC. The solid line represents the multivariable-adjusted HR, the shaded area represents the 95% CI, and the dashed line indicates the reference value of 1.0. The analysis was adjusted for the covariates included in Model 4.

### 3.5 Subgroup analyses

Subgroup analyses showed that the association between UHR and incident VC was directionally consistent across all examined subgroups (**Figure 4**). The association was statistically significant among participants aged <65 years (HR, 1.20; 95% CI, 1.06–1.36; P = 0.004), but did not reach statistical significance among those aged ≥65 years (HR, 1.23; 95% CI, 0.99–1.52; P = 0.057). Statistically significant associations were also observed among female participants (HR, 1.29; 95% CI, 1.12–1.48; P < 0.001), participants with BMI <25 kg/m² (HR, 1.29; 95% CI, 1.03–1.61; P = 0.029) or ≥25 kg/m² (HR, 1.26; 95% CI, 1.11–1.43; P < 0.001), smokers (HR, 1.28; 95% CI, 1.09–1.51; P = 0.003), and participants without hypertension (HR, 1.25; 95% CI, 1.11–1.41; P < 0.001) or with hypertension (HR, 1.79; 95% CI, 1.16–2.75; P = 0.008). The associations did not reach statistical significance among male participants (HR, 1.19; 95% CI, 0.98–1.44; P = 0.078) or nonsmokers (HR, 1.17; 95% CI, 0.99–1.37; P = 0.063). No statistically significant interactions were observed for age, sex, BMI, smoking status, or hypertension status (P for interaction = 0.908, 0.609, 0.678, 0.349, and 0.751, respectively).

**Figure 4:**
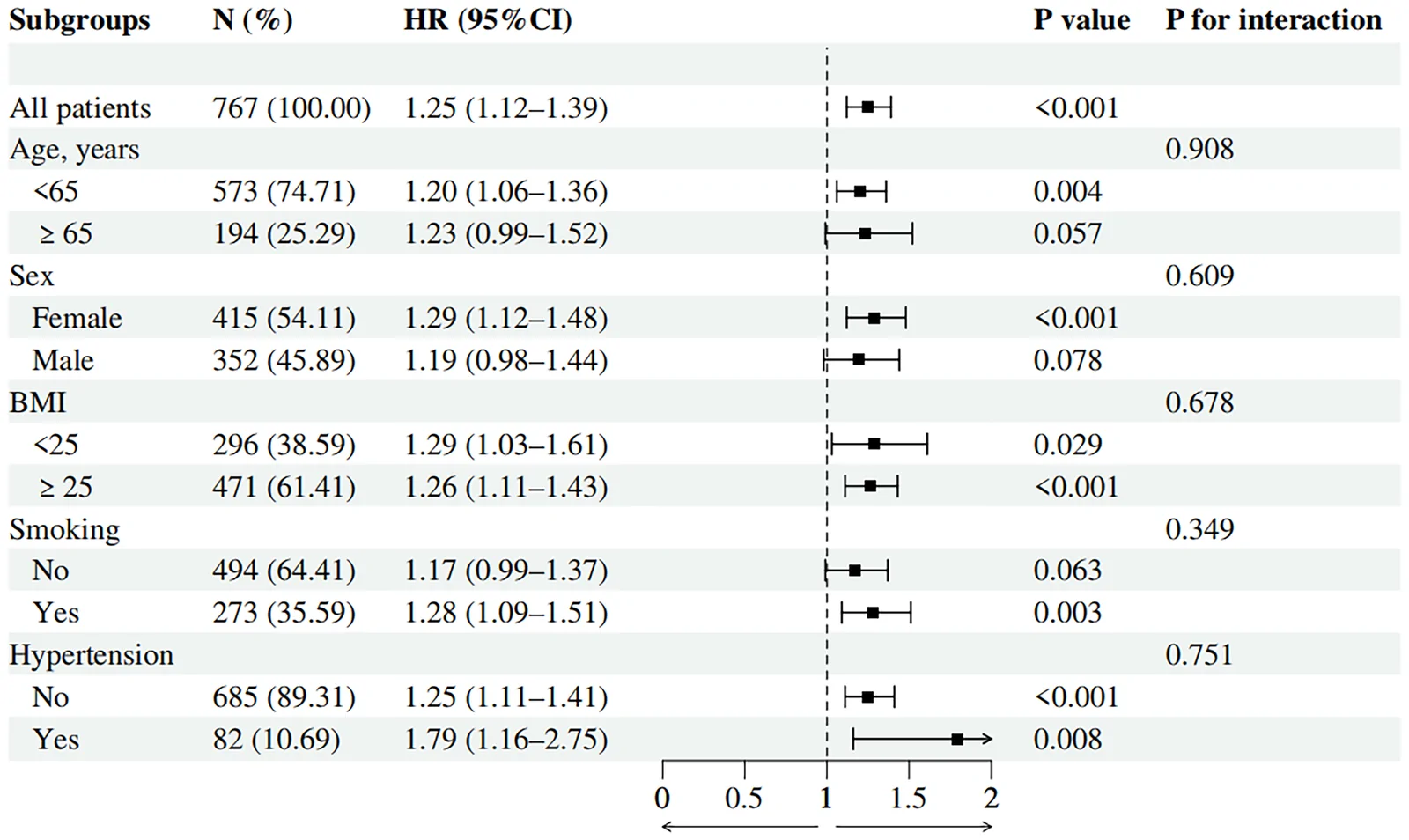
Subgroup analyses of the association between UHR and incident VC. The association was evaluated within subgroups defined by age, sex, BMI, smoking status and hypertension.

### 3.6 Sensitivity analyses

Sensitivity analyses were conducted to evaluate the robustness of the primary findings. After excluding participants who developed VC within 1 year of follow-up or those receiving lipid-lowering therapy, the association between UHR and incident VC remained consistent with the primary findings. Furthermore, the association remained robust after natural log transformation and standardization of UHR, with higher log-transformed UHR levels consistently associated with an increased risk of incident VC across all models. These findings support the robustness of the observed association between UHR and incident VC (**Supplementary Tables S4**-**S6**).

## 4 Discussion

In this retrospective cohort study of 767 hospitalized patients with T2DM and no VC at the initial assessment, 351 (45.8%) developed incident VC during follow-up. The incidence of CAC was 31.0%, which was within the previously reported range of 25.9%–37.0% in populations with T2DM, whereas the incidence of aortic calcification was 36.4%, lower than the 54% reported in a previous study (20–22). The observed incidences of VC and CAC were higher than the approximately 24% and 21%, respectively, reported in the general population (23, 24). These comparisons should be interpreted cautiously because of between-study differences in study design, follow-up duration, population characteristics, imaging protocols, anatomical sites assessed, and definitions of VC.

Multivariable Cox regression demonstrated that higher UHR was associated with an increased hazard of incident VC after adjustment for potential confounders. Quartile analysis demonstrated significantly higher hazards of incident VC in Q2–Q4 than in Q1, with significant overall association and trend tests. However, the HR for Q4 was slightly lower than that for Q3, and the Kaplan–Meier curves for Q2–Q4 substantially overlapped, suggesting that the association did not follow a strictly monotonic pattern across increasing UHR quartiles. Restricted cubic spline analysis demonstrated a significant overall association between UHR and incident VC, whereas the test for nonlinearity was not statistically significant. Exploratory two-piecewise Cox analysis suggested a potential attenuation of the association above a UHR value of approximately 11.4; however, bootstrap analysis revealed substantial uncertainty in the breakpoint estimate, and the confidence interval for the change in slope included zero. Therefore, these findings do not support the presence of a definitive threshold. No significant interactions were observed between UHR and age, sex, BMI, smoking status, or hypertension. The findings remained materially unchanged after excluding participants who developed VC within 1 year of follow-up or those receiving lipid-lowering therapy at the first hospitalization. In addition, the association remained significant after log transformation of UHR, supporting the robustness of the primary finding across different exposure specifications. Overall, these findings suggest that higher UHR was associated with a greater hazard of incident VC in patients with T2DM, and that this association remained robust after multivariable adjustment and across sensitivity analyses.

Participants who subsequently developed VC were older and had a higher prevalence of smoking, as well as higher BMI, diastolic BP, FPG, SUA, and UHR than those who remained free of VC. In DM, multiple interacting mechanisms may contribute to the development and progression of VC. These mechanisms include mitochondrial oxidative stress, the release of proinflammatory cytokines, endothelial dysfunction, dyslipidemia, and hypoxia (25). Moreover, VC likely reflects the combined effects of multiple risk factors, including older age, smoking exposure, abnormal glucose metabolism, and UA-related oxidative stress, rather than a single metabolic abnormality (3, 26, 27). Accordingly, the greater risk of VC observed at higher UHR levels may reflect both the potential contribution of UHR and the clustering of these coexisting risk factors. Nevertheless, the association between UHR and incident VC remained significant after adjustment for the principal between-group differences and other clinically relevant covariates, suggesting that the association was unlikely to be fully explained by these factors.

In 2019, Kocak et al. introduced UHR as a composite metabolic marker that may capture, at least in part, increased oxidative stress, dysregulated lipid metabolism, and impaired vascular protective capacity (14, 28–30). UHR has since been associated with adverse outcomes, including cardiovascular disease, dyslipidemia, and diabetic vascular complications (31, 32). However, direct clinical evidence linking UHR to vascular calcification remains scarce, and available studies have largely relied on cross-sectional analyses of data from the 2013–2014 NHANES cycle. Li et al. included 2,294 adults aged >40 years from the general population. After full adjustment for potential confounders, each 1-unit increase in log-transformed UHR was associated with a 0.80-point increase in the AAC score (95% CI: 0.46–1.13). The odds of AAC and severe abdominal aortic calcification (SAAC) increased by 90% (95% CI: 1.42–2.54) and 95% (95% CI: 1.37–2.77), respectively. Dose–response analyses further demonstrated positive linear or approximately linear associations of UHR with the AAC score and the odds of AAC and SAAC (12). Similarly, another analysis by Li et al. involving 2,789 individuals from the general population showed that, after multivariable adjustment, participants in the highest UHR quartile had higher odds of AAC and SAAC than those in the lowest quartile, with odds ratios (ORs) of 2.605 (95% CI: 1.760–3.855) and 2.227 (95% CI: 1.649–3.008), respectively. RCS analysis indicated an inverted L-shaped nonlinear association between UHR and AAC. The odds of AAC increased rapidly at UHR values of approximately 17.8–18.0 and subsequently reached a plateau (13). In a study of 3,009 participants, Guo et al. reported that UHR remained positively associated with the AAC score after full adjustment (β = 3.43, 95% CI: 0.45–6.41; P = 0.024). The AAC score was approximately 0.70 points higher in Q4 than in Q1. However, the association between UHR and SAAC was no longer statistically significant after full adjustment (P = 0.365), whereas RCS analysis suggested an approximately linear relationship between UHR and AAC (16). Notably, all three studies used data from the 2013–2014 NHANES cycle, and their study populations may have partially overlapped. Therefore, their findings should be interpreted as convergent evidence from a shared data source rather than as independent external validation. In contrast, the present study included patients with T2DM who showed no VC on their first chest CT and evaluated incident coronary artery and/or aortic calcification on follow-up chest CT scans. This longitudinal approach extends previous cross-sectional findings by examining incident VC with a clearly established temporal sequence, thereby providing stronger evidence regarding the temporal relationship between UHR and VC (13, 33). Furthermore, although the anatomical distribution of incident VC was recorded, the limited number of events in individual anatomical categories precluded reliable site-specific analyses of the association between UHR and calcification.

A previous systematic review and meta-analysis of eight prospective studies involving 6,521 patients with T2DM found that those with a CAC score ≥10 had a significantly higher risk of all-cause mortality or cardiovascular events than those with a CAC score <10. These findings support the utility of CAC scoring for cardiovascular risk stratification in patients with DM (34). Furthermore, in the prospective Multi-Ethnic Study of Atherosclerosis (MESA) cohort of 1,974 individuals from the general population, both AAC and CAC independently predicted hard coronary and hard cardiovascular events. However, only AAC was independently associated with cardiovascular mortality, and its association with all-cause mortality was stronger than that observed for CAC (35). Collectively, these findings suggest that incident coronary and/or aortic calcification identified in the present study may reflect the progression of subclinical vascular injury in patients with T2DM.

The dose–response patterns reported in previous studies have varied, ranging from approximately linear associations to nonlinear patterns that plateau at higher UHR levels (12, 13, 16). In the present study, RCS analysis showed a significant overall association between UHR and the hazard of incident VC (P for overall < 0.001), whereas the test for nonlinearity was not statistically significant (P for nonlinearity = 0.058). Exploratory two-piecewise Cox regression suggested that the association may be stronger at lower UHR levels and attenuated at higher UHR levels beyond a data-derived breakpoint of approximately 11.4. However, bootstrap analysis revealed substantial uncertainty in the breakpoint estimate, and the confidence interval for the change in slope included zero. Thus, although the quartile estimates, Kaplan–Meier curves, and piecewise analysis suggest that the association may not increase continuously across the full UHR range, the current data do not support a stable threshold or clinically meaningful cutoff.

The biological plausibility of the observed UHR–VC association can be better understood by considering the vascular effects of its two components, SUA and HDL-C. Elevated SUA levels may reflect altered purine metabolism and an imbalance between UA production and excretion. They are associated with metabolic syndrome and DM and may contribute to atherosclerosis through oxidative stress, inflammation, endothelial dysfunction, foam cell formation, and enhanced proliferation and migration of VSMCs (36, 37). Animal and cellular studies have shown that HUA may promote VC by inducing the osteogenic transdifferentiation of VSMCs and upregulating osteogenic genes, including Runx2, Sp7, Bglap, and Col1a1. Moreover, longer HUA exposure has been associated with greater calcium deposition in the vascular media (7, 38). However, clinical studies investigating the association between SUA and VC have yielded inconsistent findings. A longitudinal cohort study found that higher SUA levels were associated with progression from no or mild CAC at baseline to moderate CAC (39). Cross-sectional studies, however, reported either a positive dose–response relationship or a U-shaped association between SUA and AAC (6, 40). By contrast, another cross-sectional study found no statistically significant association between SUA and CAC (10). HDL contributes to cardiovascular protection through several mechanisms, including reverse cholesterol transport and anti-inflammatory, antioxidant, endothelial-protective, and antithrombotic effects (41). *In vitro* studies have shown that HDL may inhibit vascular cell calcification, partly by attenuating the effects of pro-inflammatory cytokines and oxidized lipids (42). However, clinical studies have not demonstrated a consistent independent inverse association between HDL-C and CAC. Moreover, neither HDL-C nor its subfractions have been significantly associated with the presence or severity of CAC (43–45). In contrast, Oxidized HDL (oxHDL) may promote VSMC mineralization and calcification by upregulating Interleukin-6 (IL-6), WNT-5a, NF-κB p65, and Osterix, whereas reductions in oxHDL levels have been associated with slower CAC progression (2, 46). Taken together, these findings suggest that UHR may capture complementary information on UA-related metabolic and inflammatory stress and HDL-mediated vascular protection, thereby reflecting a broader pro-calcific vascular milieu associated with incident VC.

Furthermore, UHR may reflect, at least in part, the combined effects of multiple metabolic abnormalities and their related pathophysiological processes. Previous studies have shown that higher UHR is associated with insulin resistance in patients with T2DM (47), while insulin resistance may contribute to the development and progression of vascular calcification (48). In addition, higher UHR has been associated with nonalcoholic fatty liver disease (NAFLD) (49), which, in turn, has been linked to the development and progression of coronary artery calcification (50).

In the context of T2DM, hyperglycemia, oxidative stress, and chronic inflammation may promote the osteogenic transdifferentiation of VSMCs, thereby contributing to a pro-calcific vascular microenvironment (51–53). Therefore, the observed association between UHR and incident VC may partly reflect a pro-calcific pathophysiological milieu characterized by multiple metabolic and inflammatory abnormalities, rather than the effect of a single biological pathway.

In this study, the association between UHR and incident VC was statistically significant in women but did not reach statistical significance in men, although the interaction by sex was not statistically significant. Although this finding does not indicate significant effect modification by sex, sex hormones may influence the observed association through their effects on SUA and HDL-C metabolism. Previous studies have suggested that estradiol (E2) and progesterone (P4) may exert cardioprotective effects in women of reproductive age, with higher estrogen levels being associated with a lower risk of cardiovascular disease (54, 55). In addition, SUA levels tend to increase after menopause, and higher SUA has been reported to independently predict cardiovascular events in postmenopausal women (13, 56). Sex hormones may also influence this association through their effects on HDL metabolism. One study reported an inverse association between HDL particle concentrations and coronary artery calcification among women with higher estradiol levels, with a significant interaction (P for interaction = 0.007) (57). Another study found that higher concentrations of large HDL particles were associated with a lower likelihood of coronary artery calcification, although this association was not independent of HDL-C levels (58). Nevertheless, because the interaction by sex was not statistically significant in the present study, these potential biological explanations should be considered exploratory and interpreted cautiously.

A key strength of this study is the inclusion of patients without evidence of VC on baseline chest CT and the longitudinal ascertainment of incident coronary and/or aortic calcification, which addresses the limitations of previous cross-sectional studies in establishing temporal relationships. The consistency of findings across multiple analytical approaches further supports the robustness of the observed association. Because UHR can be readily calculated from routinely measured SUA and HDL-C levels, it is easy to obtain, inexpensive, and potentially applicable in routine clinical settings.

Several limitations should be acknowledged. First, this single-center retrospective study included a selected population of hospitalized patients with T2DM who underwent repeated chest CT examinations, potentially limiting generalizability and introducing selection or surveillance bias. Although CT indications were comparable between participants with and without incident VC, follow-up imaging was not performed according to a standardized schedule. Consequently, the exact timing of calcification onset could not be determined, and events occurring outside the study center may have been missed. Second, UHR was measured only at the first hospitalization and therefore may not capture longitudinal changes in SUA or HDL-C. Although SUA and HDL-C were also analyzed separately, whether UHR provides additional value beyond its individual components remains uncertain. Third, residual confounding cannot be excluded because detailed information on specific antihypertensive and glucose-lowering therapies was not comprehensively available. Fourth, VC was assessed qualitatively on routine chest CT without standardized quantitative calcium scoring, which may have reduced sensitivity for mild calcification and precluded assessment of calcification burden, severity, and progression. In addition, site-specific analyses were constrained by the limited number of events within individual anatomical categories.

## 5 Conclusion

In this retrospective cohort study of 767 hospitalized patients with T2DM without visible VC on baseline chest CT, higher UHR was independently associated with an increased risk of incident coronary and/or aortic calcification after multivariable adjustment. Each 1-SD increase in UHR was associated with a 24.5% higher hazard of incident VC. Although significant overall and median-based trends were observed across UHR quartiles, the quartile-specific estimates did not follow a strictly monotonic pattern. Exploratory analyses suggested attenuation of the association at higher UHR levels but did not support a stable threshold. Overall, this study extends previous cross-sectional evidence by providing longitudinal evidence in patients with T2DM and supports further evaluation of UHR as a potential marker of VC risk. Multicenter prospective studies incorporating repeated measurements of UHR and its components, standardized site-specific imaging protocols, and comprehensive assessment of clinical and pharmacological factors are warranted to validate these findings and further clarify the role of UHR in VC risk assessment.

## Data Availability

The datasets analyzed in this study are not publicly available to protect patient privacy and confidentiality. Requests for data access should be directed to the corresponding author, Xuhan Liu (xuhanliu281277@163.com). Data sharing is subject to applicable ethical, institutional, and data protection requirements, including approval from Central Hospital of Dalian University of Technology, where required.
