# Supplementary material for: Uric acid-to-high-density lipoprotein cholesterol ratio and incident vascular calcification in patients with type 2 diabetes mellitus: a retrospective cohort study
Source: Front Endocrinol (Lausanne). 2026 Oct 9;17:1964678. doi: 10.3389/fendo.2026.1964678 (PMC13700012; doi:10.3389/fendo.2026.1964678)
Supplement: Supplementary file 1 [file DataSheet1.docx]

**Supplemental files**

**Uric Acid-to-High-Density Lipoprotein Cholesterol Ratio and Incident Vascular Calcification in Patients With Type 2 Diabetes Mellitus: A Retrospective Cohort Study**

**Taoyuan He^1, 2†^, Xin Wang^1†^, Guosheng Li^3†^, Xue Liang^4†^, Yuntong Liu^5^, Bing Wang^1^, Yudan Liu^6^, Wei Zhao^1^, Zhu Zhu^1^, Xue Yao^7, 8^, Yue Xiao^9^, Xuhan Liu^1*^**

^1^Department of Endocrinology, Central Hospital of Dalian University of Technology, 826 Xinnan Road, Shahekou District, Dalian City, Liaoning Province, 116033, China.

^2^General Practice Ward/International Medical Center Ward, General Practice Medical Center, West China Hospital, Sichuan University, Chengdu, China

^3^Laboratory Pathology Department, Ningbo Clinical Pathology Diagnosis Center, Ningbo, 315021, China.

^4^Department of Respiratory Medicine, Yanhu District People's Hospital of Yuncheng, Yuncheng City, 044000, China

^5^China-Japan Friendship Hospital (Institute of Clinical Medical Sciences), Chinese Academy of Medical Sciences & Peking Union Medical College, Beijing 100730, China

^6^Department of Neuroendocrine Pharmacology, School of Pharmacy, China Medical University, Shenyang, 210009, China.

^7^Graduate School, Dalian Medical University, Liaoning, China.

^8^Department of Internal medicine, Traditional Chinese Medicine Hospital of Dalian Pulandian District, Liaoning, China.

^9^The Second Affiliated Hospital of Dalian Medical University, Dalian Medical University, Dalian 116041, China

*Correspondence: Xuhan Liu, xuhanliu281277@163.com

^†^These authors contributed equally to this work.

**Table of contents**

**Table S1** Indications for baseline chest CT examinations.

**Table S2** Indications for outcome-defining follow-up chest CT examinations.

**Table S3** Associations of UHR and its components with incident VC.

**Table S4** Sensitivity analysis excluding participants who developed VC within 1 year of follow-up.

**Table S5** Sensitivity analysis after excluding participants treated with lipid-lowering therapy.

**Table S6** Sensitivity Analysis of the Association Between Log-Transformed UHR and Incident VC.

**Figure S1** Schoenfeld residual plots assessing the proportional hazards assumption for the Cox model with UHR modeled per 1-SD increase.

**Figure S2** Schoenfeld residual plots assessing the proportional hazards assumption for the Cox model with UHR modeled in quartiles.

**Table S1** Indications for baseline chest CT examinations.

| **Indication** | **Total** | **Non-VC** | **VC** |
| --- | --- | --- | --- |
| Respiratory symptoms or suspected pulmonary disease | 333 (43.4) | 174 (41.8) | 159 (45.3) |
| Follow-up of previously identified thoracic abnormalities | 205 (26.7) | 110 (26.4) | 95 (27.1) |
| Preoperative or perioperative assessment | 61 (8.0) | 35 (8.4) | 26 (7.4) |
| Routine or other non-acute clinical evaluation | 124 (16.2) | 73 (17.5) | 51 (14.5) |
| Other or unclear indications | 44 (5.7) | 24 (5.8) | 20 (5.7) |

Data are presented as n (%). Two investigators independently classified the indications for baseline chest CT examinations, with high interrater agreement (Cohen’s κ = 0.886). The overall distribution of baseline CT indications did not differ significantly between participants with and without incident VC (P = 0.759). P values were calculated using the chi-square test or Fisher’s exact test, as appropriate.

**Table S2** Indications for outcome-defining follow-up chest CT examinations.

| **Indication** | **Total** | **Non-VC** | **VC** |
| --- | --- | --- | --- |
| Respiratory symptoms or suspected pulmonary disease | 293 (38.2) | 164 (39.4) | 129 (36.8) |
| Follow-up of previously identified thoracic abnormalities | 289 (37.7) | 158 (38.0) | 131 (37.3) |
| Preoperative or perioperative assessment | 53 (6.9) | 24 (5.8) | 29 (8.3) |
| Routine or other non-acute clinical evaluation | 93 (12.1) | 45 (10.8) | 48 (13.7) |
| Other or unclear indications | 39 (5.1) | 25 (6.0) | 14 (4.0) |

Data are presented as n (%). For participants with incident VC, the outcome-defining follow-up CT was the first examination showing incident VC; for those who remained free of VC, it was the last available chest CT examination. Two investigators independently classified the indications for the outcome-defining follow-up CT examinations, with high interrater agreement (Cohen’s κ = 0.881). The overall distribution of follow-up CT indications did not differ significantly between participants with and without incident VC (P = 0.298). The P value was calculated using the chi-square test or Fisher’s exact test, as appropriate.

**Table S3** Associations of UHR and its components with incident VC.

| **Variable** | **Model 1** | | **Model 2** | | **Model 3** | | **Model 4** | |
| --- | --- | --- | --- | --- | --- | --- | --- | --- |
|  | **HR (95% CI)** | ***P* value** | **HR (95% CI)** | ***P* value** | **HR (95% CI)** | ***P* value** | **HR (95% CI)** | ***P* value** |
| UHR | 1.194 (1.080–1.319) | <0.001 | 1.231 (1.113–1.362) | <0.001 | 1.235 (1.112–1.371) | <0.001 | 1.245 (1.117–1.387) | <0.001 |
| SUA | 1.266 (1.151–1.394) | <0.001 | 1.228 (1.113–1.355) | <0.001 | 1.112 (0.999–1.238) | 0.052 | 1.073 (0.959–1.201) | 0.220 |
| HDL-C | 1.005 (0.903– 1.120) | 0.922 | 0.975 (0.873–1.088) | 0.648 | 0.954 (0.852–1.068) | 0.415 | 0.933 (0.831–1.048) | 0.244 |

HRs represent the relative hazard of incident vascular calcification associated with each 1-SD increase in UHR, SUA, or HDL-C. Model 1 was unadjusted. Model 2 was adjusted for age and sex. Model 3 was further adjusted for alcohol use, smoking status, family history, hypertension, BMI, FPG, HbA1c, DM duration, and eGFR. Model 4 was additionally adjusted for TG, ALP, Mg, Ca, insulin therapy, and lipid-lowering therapy.

**Table S4** Sensitivity analysis excluding participants who developed VC within 1 year of follow-up.

| **Variable** | **Model 1** |  | **Model 2** |  | **Model 3** |  | **Model 4** |  |
| --- | --- | --- | --- | --- | --- | --- | --- | --- |
|  | **HR (95% CI)** | ***P* value** | **HR (95% CI)** | ***P* value** | **HR (95% CI)** | ***P* value** | **HR (95% CI)** | ***P* value** |
| **UHR** |  |  |  |  |  |  |  |  |
| Per 1 SD increase | 1.255 (1.110–1.419) | <0.001 | 1.298 (1.144–1.472) | <0.001 | 1.310 (1.152–1.490) | <0.001 | 1.320 (1.153–1.511) | <0.001 |
| Quartiles 1 | Ref |  | Ref |  | Ref |  | Ref |  |
| Quartiles 2 | 1.837 (1.214–2.782) | 0.004 | 1.943 (1.281–2.949) | 0.002 | 1.780 (1.167–2.717) | 0.007 | 1.703 (1.115–2.602) | 0.014 |
| Quartiles 3 | 1.908 (1.266–2.873) | 0.002 | 2.051 (1.358–3.099) | <0.001 | 2.043 (1.351–3.091) | <0.001 | 1.976 (1.304–2.996) | 0.001 |
| Quartiles 4 | 2.101 (1.413–3.125) | <0.001 | 2.262 (1.517–3.374) | <0.001 | 2.184 (1.460–3.266) | <0.001 | 2.124 (1.410–3.199) | <0.001 |
| *P* for trend |  | <0.001 |  | <0.001 |  | <0.001 |  | <0.001 |

Model 1 was unadjusted.

Model 2 was adjusted for age and sex.

Model 3 was further adjusted for alcohol use, smoking status, family history, hypertension, BMI, FPG, HbA1c, DM duration, and eGFR.

Model 4 was additionally adjusted for TG, ALP, Mg, Ca, insulin therapy, and lipid-lowering therapy.

**Table S5** Sensitivity analysis after excluding participants treated with lipid-lowering therapy.

| **Variable** | **Model 1** |  | **Model 2** |  | **Model 3** |  | **Model 4** |  |
| --- | --- | --- | --- | --- | --- | --- | --- | --- |
|  | **HR (95% CI)** | ***P* value** | **HR (95% CI)** | ***P* value** | **HR (95% CI)** | ***P* value** | **HR (95% CI)** | ***P* value** |
| **UHR** |  |  |  |  |  |  |  |  |
| Per 1 SD increase | 1.193 (1.079–1.319) | <0.001 | 1.228 (1.110–1.359) | <0.001 | 1.236 (1.113–1.373) | <0.001 | 1.247 (1.119–1.390) | <0.001 |
| Quartiles 1 | Ref |  | Ref |  | Ref |  | Ref |  |
| Quartiles 2 | 1.648 (1.177–2.305) | 0.004 | 1.728 (1.233–2.421) | 0.001 | 1.619 (1.153–2.276) | 0.005 | 1.582 (1.124–2.225) | 0.008 |
| Quartiles 3 | 1.847 (1.328–2.568) | <0.001 | 1.993 (1.431–2.778) | <0.001 | 1.917 (1.374–2.674) | <0.001 | 1.885 (1.347–2.637) | <0.001 |
| Quartiles 4 | 1.816 (1.313–2.512) | <0.001 | 1.957 (1.412–2.712) | <0.001 | 1.855 (1.334–2.580) | <0.001 | 1.857 (1.328–2.595) | <0.001 |
| *P* for trend |  | <0.001 |  | <0.001 |  | <0.001 |  | <0.001 |

Model 1 was unadjusted.

Model 2 was adjusted for age and sex.

Model 3 was further adjusted for alcohol use, smoking status, family history, hypertension, BMI, FPG, HbA1c, DM duration, and eGFR.

Model 4 was additionally adjusted for TG, ALP, Mg, Ca, insulin therapy, and lipid-lowering therapy.

**Table S6** Sensitivity Analysis of the Association Between Log-Transformed UHR and Incident VC.

| **Variable** | **Model 1** |  | **Model 2** |  | **Model 3** |  | **Model 4** |  |
| --- | --- | --- | --- | --- | --- | --- | --- | --- |
|  | **HR (95% CI)** | ***P* value** | **HR (95% CI)** | ***P* value** | **HR (95% CI)** | ***P* value** | **HR (95% CI)** | ***P* value** |
| Log (UHR) per 1-SD increase | 1.228 (1.102–1.368) | <0.001 | 1.272 (1.140–1.419) | <0.001 | 1.272 (1.136–1.424) | <0.001 | 1.282 (1.141–1.441) | <0.001 |

Model 1 was unadjusted.

Model 2 was adjusted for age and sex.

Model 3 was further adjusted for alcohol use, smoking status, family history, hypertension, BMI, FPG, HbA1c, DM duration, and eGFR.

Model 4 was additionally adjusted for TG, ALP, Mg, Ca, insulin therapy, and lipid-lowering therapy.

**Figure S1** Schoenfeld residual plots assessing the proportional hazards assumption for the Cox model with UHR modeled per 1-SD increase.

**Figure S2** Schoenfeld residual plots assessing the proportional hazards assumption for the Cox model with UHR modeled in quartiles.
